# Endothelial progenitor cell biology in disease and tissue regeneration

**DOI:** 10.1186/1756-8722-4-24

**Published:** 2011-05-24

**Authors:** Andrea L George, Pradeep Bangalore-Prakash, Shilpi Rajoria, Robert Suriano, Arulkumaran Shanmugam, Abraham Mittelman, Raj K Tiwari

**Affiliations:** 1 Department of Microbiology and Immunology, New York Medical College, Valhalla, New York, USA

**Keywords:** Endothelial Progenitor Cells, Neovascularization, Estrogen, Cancer, Proangiogenic proteins, Inflammation, Tumor Microenvironment, Cluster of Differentiation Antigens, Chemokines

## Abstract

Endothelial progenitor cells are increasingly being studied in various diseases ranging from ischemia, diabetic retinopathy, and in cancer. The discovery that these cells can be mobilized from their bone marrow niche to sites of inflammation and tumor to induce neovasculogenesis has afforded a novel opportunity to understand the tissue microenvironment and specific cell-cell interactive pathways. This review provides a comprehensive up-to-date understanding of the physiological function and therapeutic utility of these cells. The emphasis is on the systemic factors that modulate their differentiation/mobilization and survival and presents the challenges of its potential therapeutic clinical utility as a diagnostic and prognostic reagent.

## Introduction

As a new decade of cancer research begins, many of the same problems in investigating tumor growth and metastasis remain. Much of the difficulty is due to the heterogeneity of not only the tumor types, but the cellular environment of the individual tumors themselves. All cancers though still go through specific initiation, promotion, and progression phases. The initiation events are varied from endogenous metabolites to exogenous insults while the tumor microenvironment in part dictates the promotion and progression phases. The unanswered questions of why some tumors remain benign while others become malignant, why some only grow at their primary foci while others rapidly metastasize, and why some are susceptible to chemotherapeutics while others remain resistant is still an enigma. These differences have lead researchers to develop new strategies of cancer treatment aimed at the body's normal physiological processes that tumors are able to manipulate to their own end.

One recent strategy that has emerged in cancer research involves targeting of tumor associated blood vessels which provide growing tumors with oxygenated blood and growth factors necessary for maintenance and metastasis. The uncontrolled growth of tumors leads to formation of a hypoxic tumor microenvironment leading to a proangiogenic signalling cascade. Initial work was focused on tumor induced angiogenesis, or sprouting of existing vasculature toward the tumor. However, recent research has identified a novel mechanism in vasculature development known as vasculogenesis, or the formation of new vessels from bone marrow derived progenitor cells rather than sprouting or elongation of existing vessels. Neovasculogenesis is due, in part, to bone marrow-derived endothelial progenitor cells (BM-EPCs) which are released from the marrow and home to sites of blood vessel formation.

While the rapid expansion of cells leads to activation of neovascularization, the process relies on the formation of a hypoxic, and thus inflammatory, tumor microenvironment that signals not only for progenitor but also immunomodulatory cell migration. Secretion of proangiogenic as well as both pro and anti-inflammatory cytokines by these modulating cells also influences the genetic and phenotypic characteristics of tumor cells. Such cytokines include IL-1 and TGF-β which lead to an epithelial to mesenchymal transition (EMT) during which tumor cells downregulate epithelial markers including E-cadherin and upregulate mesenchymal markers as well as transcription factors like SNAIL and TWIST increasing their metastatic propensity [1-3]. Inflammation, also induced by cytokines secreted by infiltrating macrophages, alters the tumor cell epigenome and modulation of proangiogenic proteins [4]. Tumor cell development is comparable to a "wound" that never heals in which a steady inflammatory environment is propagated. Future work must also be directed toward the influx of immunomodulating cells and their cytokine profile.

We however have identified another mechanism of BM-EPC induced vasculogenesis, in which progenitor cells contribute to the development of breast tumor vessel formation in an estrogen dependent manner [5]. Indeed, clinically circulating EPCs are being correlated with increased tumor growth and since they home to tumor sites are being targeted as potential Trojan Horses for specific gene therapy delivery [6]. Identification of this novel cell type's role in neovasculogenesis may provide researchers a common target for anti-tumor therapy directed against the tumor and the tumor microenvironment. This review, while focusing on the difference between angiogenesis and vasculogenesis, the characteristics of the bone marrow derived progenitor cells that contribute to neovasculogenesis, and the factors that modulate them, places the process of neovasculogenesis as a necessary modulator of the tumor microenvironment capable of promoting a subset of tumor cells which are responsible for tumor progression.

## Angiogenesis vs. Vasculogenesis

Angiogenesis is the formation of new vessels from existing vasculature by two distinct methods termed sprouting and non-sprouting angiogenesis. Sprouting angiogenesis (SA) occurs when endothelial cells migrate and divide off the existing vessels and fusion of vacuoles within the endothelial cells creates the vascular lumen [7]. Migration of these cells relies on a source of proangiogenic stimuli as well as proteases that degrade the basement membrane which allows mobilization and proliferation of endothelial cells that later form sprouts. Non-sprouting angiogenesis, or intussesceptive angiogenesis (IA), occurs via splitting of an already existing vessel into two by formation of transcapillary pillars followed by vascular myogenesis, although the exact mechanism is poorly understood [7]. Angiogenesis is necessary during embryonic development but also plays important roles throughout postnatal life in wound healing, tissue ischemia, and tumor vasculature formation and is now a major therapeutic target in cancer treatment.

However, recent studies have shown that a mechanism different from angiogenesis exists for formation of vessels in adults called postnatal vasculogenesis or neovascularization. During vasculogenesis precursor cells from adult bone marrow are mobilized into circulation in response to various signals and home to the source where they differentiate into mature endothelial cells, assisting in the ongoing vascular development [8]. Neovascularization is a critical process for revascularization of ischemic tissues and wound healing but plays a role pathologically as it can be induced by cancers to aid in tumor growth and metastasis, and can also be seen in conditions like diabetic retinopathy and retinopathy of prematurity [8]. The bone marrow precursor cells that aid in neovascularization are known as endothelial progenitor cells.

## EPCs: Physiological and Biological Functions

Endothelial progenitor cells (EPCs) are bone marrow derived cells that can be found in the peripheral and umbilical cord blood and were first isolated using magnetic micro beads by Asahara [9]. Studies have shown that the term 'EPCs' cannot be used to define a single cell type but rather should be used to refer to multiple cell types capable of differentiating into the endothelial lineage [10]. First, they are considered derivatives of hemangioblasts and express CD34, VEGFR-2 and CD133 on their surface (Table 1). CD133, a transmembrane, 120 kDa glycoprotein, is expressed by EPCs but not by mature endothelial cells. These adult EPCs and the embryonic angioblasts share similar characteristics as both are derived from the hemangioblast precursors and both have the capacity to home to the periphery where they proliferate and differentiate into mature endothelial cells. Second, EPCs are considered one subset of cells derived from bone marrow multipotent adult progenitor cells (MAPCs). MAPCs also express CD133 and VEGFR-2 but lack CD34 or vascular endothelial cadherin expression [10]. *In vitro *experiments on MAPCs have shown that they differentiate into mature endothelial cells when grown in a serum-free media with VEGF (Table 1). Lastly, the myelo/monocytic cells, also derived from the bone marrow, can differentiate into EPCs [10]. The myelo/monocytic cells express CD14 on their surface and form mature endothelial cells positive for vWF, VEGFR-2, and CD45 (common leukocyte antigen) expression when cultured (Table 1). Irrespective of their origin, EPCs in general have the functional ability to take up acetylated LDL, and bind to *Ulex europaeus *agglutinin 1 (UEA1) [11]. Hence, *in vivo *three groups of progenitors have been found to differentiate into mature endothelial cells, the hemangioblasts, the MAPCs and the myelo/monocytic cells. Two groups of EPCs have been defined in *in vitro *models, the early EPCs, which are derived from the monocytes and have surface expression of CD45, CD14, CD11b and CD11c, and the late EPCs, which are believed to be a subset of CD14^- ^CD34^- ^KDR^-^(kinase insert domain protein receptor) cells that do not express CD45 or CD14 [12].

**Table 1:** Cell surface markers that functionally define EPCs

Surface Markers	Function	Cell Expression
CD34	Glycoprotein important for cell-cell adhesion, maintenance of stem cells in bonemarrow, mediates attachment of leukocytes to high endothelial venules [57]	Hemangioblasts, Endothelial Progenitor Cells, Vascular Endothelial Cells [10]

VEGFR-1 (Flt1)	Tyrosine kinase receptor for VEGF A and B, important for endothelial cell assembly into vessels [58]	MAPC, Myelo/Monocytic Progenitors, Vascular Endothelial Cells [58]

VEGFR-2 (Flk1, KDR)	Tyrosine kinase receptor for VEGF A,C,D,&E, critical for hematopoietic and endothelial cell development, principal mediator of VEGF-A mitogenic and pro-migration ability [59]	Hemangioblasts, Endothelial Progenitor Cells, MAPC, Myelo/Monocytic Cells, Vascular Endothelial Cells, Lymphatic Endothelial Cells [10]

CD133 (Prominin 1)	Membrane glycoprotein, function unknown, serves as a marker for hematopoietic and endothelial progenitor cells [60]	Hematopoietic Cells, Endothelial Progenitor Cells [10]

CD45	Protein tyrosine phosphatase, important for lymphocyte activation via LCK and FYN [61]	Cells of Hematopoietic System [61]

VE-cadherin	Calcium dependent glycoprotein, intercellular junction protein necessary for proper vascular development [62]	Mature Endothelial Cells [62]

vWF	Secreted glycoprotein important for platelet aggregation [63]	Produced by Endothelial Cells and Megakaryocytes, Stored in Platelets [10,63]

Studies of EPC modulation and function require their isolation and expansion. EPCs are obtained from *ex vivo/in vitro *culture of unfractionated peripheral blood mononuclear cells (MNCs) or by direct flushing of bone marrow and expansion in endothelial specific media. Only two different cell types have been isolated from the cultures so far, the endothelial cell-like cells (EC-like cells), and the endothelial outgrowth cells (EOCs). These two cell types have few similarities; they can effect neovascularization *in vivo*, take up LDL by binding UEA-1 lectin and have similar surface markers such as CD31, vWF [13,14]. However, the EC-like cells are derived from CD45^+ ^hematopoietic lineage cells, they are spindle shaped and are generated after 4-21 days in culture, they have a low proliferative potential and do not produce vascular tubes *in vitro*. *In vivo *they have myeloid progenitor cell activity and differentiate into macrophages but they do not form vessels [13,14]. Although they are unable to form vessels directly, they have an indirect paracrine effect on angiogenesis by secreting angiogenic factors locally. Hence, these cells are not considered true EPCs but can be referred to as 'Angiogenic cells' [13]. The EOCs, unlike the EC-like cells, originate from CD45^- ^CD133^- ^CD34^+ ^cells and do not have hematopoietic surface markers. EOCs express CD31, CD34, CD105, CD146, VE-Cadherin, and VEGFR-2 on their surface. In cultures they are polygonal cells and appear after 7 days, they are highly proliferative, and they do not differentiate into hematopoietic cells. The EOCs can form vessels both *in vitro *and *in vivo *[13].

To aid in neovasculogenesis, EPCs mobilize from the bone marrow in response to endogenous or exogenous signals and home to peripheral tissue sites. Their surface receptor P-selectin glycoprotein ligand-1(PSGL-1) interacts with P-selectin and E-selectin expressed on endothelial cells, followed by autocrine and paracrine activation of EPCs resulting in differentiation or transdifferentiation, proliferation and vascular growth [12]. β 2 integrins (LFA-1, Mac-1) and β 1 integrin also mediate homing of the EPCs to the periphery and β 2 integrin helps in the arrest and migration of EPCs across the endothelial cells [15]. The physiological function of circulating EPCs is to maintain vascular integrity which is also crucial in the pathogenesis of various diseases with vascular insult. The vasculogenic potential of EPCs is also exploited by tumors by recruiting EPCs to facilitate their growth and metastasis [12].

**Figure 1:**
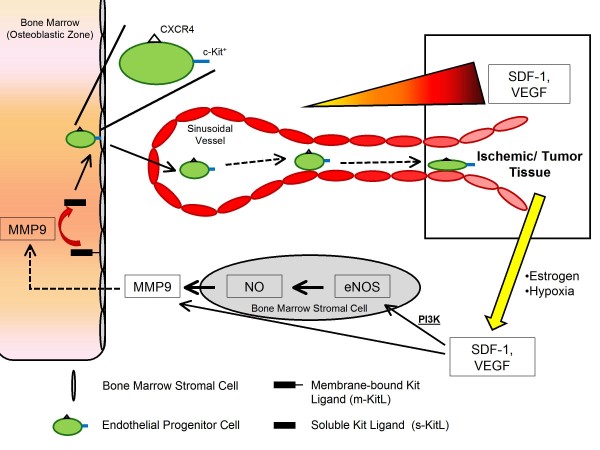
**Trafficking of EPCs to ischemic/tumor tissues as directed by major cytokine/chemokine expression**. Endothelial progenitor cell homing from the bone marrow niche to sites of neovasculogenesis is dependent a cytokine/chemokine gradient. The cellular stress induced by ischemic and tumor tissue leads to the release of a number of pro-angiogenic factors, including VEGF. VEGF stimulation of stromal cells leads to an increase in eNOS and NO production, leading to MMP-9 secretion. MMP-9 then converts m-KitL to s-KitL aiding in the release of EPCs from bone marrow stromal cells. The EPCs then migrate toward the angiogenic gradient via chemokine receptors including CXCR-4 and VEGFR-2.

EPCs are not only involved in physiological neovascularization but also involved in wound healing, tissue regeneration in ischemia (e.g. myocardial ischemia, limb ischemia), tissue remodelling (Diabetes mellitus and Heart failure) and neovascularization and growth of tumors [16]. EPCs are mobilized from the bone marrow in response to paracrine signals generated by ischemic tissue and tumor cells including GM-CSF and VEGF, which play a critical role in mobilization of EPCs to ischemic tissues and tumors. Hypoxia in tumors and ischemic tissues mediate EPC recruitment by activation of HIF-1 which leads to increased synthesis of a potent angiogenic factor VEGF. Also growing tumors secrete a number of other factors like fibroblast growth factor (FGF), SDF-1, osteopontin, CCL2 and CCL5 which help in EPC mobilization [17]. EPCs are then released into circulation by activation of MMP-9 which degrades the extracellular matrix and transforms membrane-crossing Kit ligand (mKitL) to solubility Kit ligand (sKitL) in the bone marrow [18,19] (Figure 1). The physiological function of circulating EPCs is to maintain vascular integrity which is also crucial in the pathogenesis of various diseases with vascular insult. The vasculogenic potential of EPCs is also exploited by tumors by recruiting EPCs to facilitate their growth and metastasis [12]. The tumor microenvironment plays a major role in activating circulating EPCs and mediating neovascularization and stressors in the tumor microenvironment such as hypoxia, glucose deprivation, and reactive oxygen species upregulate transcription of angiogenic factors like VEGF, SDF-1, MCP-1, and erythropoietin in EPCs [12,20,21]. Also CCL11 mediates tumor angiogenesis by recruitment and activation of eosinophils which secrete angiogenic factors [22].

Tumor growth has an avascular and vascular phase, and it is in the avascular phase of tumor growth and ischemic tissue that hypoxia induced EPC mobilization is active [20,21]. The EPCs recruited to the tumor or ischemic sites have a direct structural function by forming the vessel or an indirect paracrine effect by secreting angiogenic factors. The role of EPCs in tumor neovascularization was studied in an Id1 +/- Id3-/- mouse model which is tumor resistant and has defective angiogenesis, where transplantation of wild type bone marrow to the mutant mice restored tumor angiogenesis and growth. In the same study they also found that both VEGFR1 and VEGFR2 are required for tumor growth and blocking these receptors together completely abolishes tumor growth [23]. Mobilization and incorporation of EPCs in tumor vessels varies with the tumor type, tumor stage and tumor treatment. Studies on different types of tumors and EPCs have shown an increase in the circulating EPC population in lymphomas, leukemia, hepatocellular carcinoma, and colon cancer. Because of this EPCs have a diagnostic, therapeutic and prognostic potential in cancers. EPCs can thus act as biomarkers of tumor development and/or progression and can be studied by injection of labelled AC133+ cells and tracking it with MRI. EPCs are known to home to tumor tissues, and this property allows their use as a therapeutic delivery vehicle in combination with targeted anti-angiogenic or cytotoxic effects. EPCs are also used as gene delivery vehicles to tumor tissues [20,24]. The physiological significance of EPCs is varied and is of relevance in both normal and tumor tissue regeneration. Clinical exploitation of these cells is critically dependent on the biology of its modulators both systemic and cell derived soluble proteins.

## Modulation of EPC Functions

EPC homing relies on creation of a gradient of endogenous proteins. One of the best studied is vascular endothelial growth factor (VEGF), a homodimeric glycoprotein with a molecular weight of 45 kDa which is synthesized by normal cells and upregulated by hypoxia. VEGF is not only secreted locally where it has paracrine like effects but is also secreted into circulation and acts as a hormone [25]. Under hypoxic conditions transcription factors like Hypoxia Inducible Factor - 1 (HIF-1) are activated leading to increased transcription of VEGF [24]. VEGF stimulates VEGFR1 and VEGFR2 receptors present on endothelial and hematopoietic stem cells and activates matrix metalloproteinase - 9 (MMP-9) which in turn cleaves and activates Kit ligand (KitL) and induces proliferation and migration of EPCs and hematopoietic cells [26].

The proangiogenic protein angiogenin also plays a role in EPC function. Angiogenin is a 14-kDa protein that binds and activates endothelial cells leading to proliferation and migration and has ribonucleolytic activity. Angiogenin also translocates to the nucleus of cells, which is necessary for other proteins, including VEGF, to exert their proangiogenic effects [27]. Angiogenin may bind with follistatin, another angiogenic protein that in an *in vivo *model was found to increase the number of tumor associated capillaries but not tumor size [28,29].

Another family of angiogenic factors is the Angiopoietins (Ang-1, 2), 57 kDa proteins that regulate both neoplastic and non neoplastic neovasculogenesis in the embryo and post natal life and mitigate their effects by binding cognate tyrosine kinase receptors (Tie-1 and Tie-2). Ang-1 can activate the receptor Tie-2 and lead to downstream activation of the phosphatidylinositol 3'-kinase/Akt prosurvival pathway in endothelial cells. *In vivo *however, studies on Ang-1 have showed that over expression of Ang-1 in tumors decreases tumor neovascularization and tumor size [30]. The function of Ang-2 still remains controversial, as early models suggested Ang-2 was a functional antagonist of Ang-1, however, a role of Ang-2 in vessel sprouting has been identified [31,32].

Cytokines also promote EPC mobilization to the periphery. Granulocyte-colony stimulating factor (G-CSF) and granulocyte monocyte-colony stimulating factor (GM-CSF) are glycoproteins which stimulate production of granulocytes in the bone marrow, and also influence the proliferation, differentiation, and migration of bone marrow EPCs [33]. Another cytokine that may play a role in EPC modulation includes IL-8. Binding of IL-8 to human umbilical vein endothelial cells (HUVECs) that express the receptors CXCR1 and CXCR2 lead to endothelial cell proliferation and capillary tube formation *in vitro *[34]. Further, in acute myocardial infarction, IL-8 was associated with an increase in circulating CD133+ cells [35]. Taken together with the fact that breast cancer patients in higher stages had significantly more IL-8 mRNA may shed light on a novel role of IL-8 on progenitor cell mediated neovascularization [36].

Chemokines and their receptors are involved in EPC migration and differentiation as well. CCR2 is a chemokine receptor expressed on the surface of EPCs and vascular smooth muscle cells (VSMCs) that mediates chemotaxis to areas of endothelial denudation, which secrete monocyte chemoattractant protein-1 (MCP-1/CCL2), leading to angiogenesis [37]. EPCs also express another chemokine receptor CCR5 which binds its ligand RANTES/CCL5 and plays an important role in atherogenesis and vascular remodelling [37]. CXCL12 or stromal cell derived factor - 1α (SDF-1α) is another chemokine responsible for EPC mobilization and also recruitment along hypoxic gradients via the CXCR4 receptor. During tumor growth, hypoxic regions stimulate the transcription factor hypoxia inducible factor 1 (HIF-1) leading to transcription of proangiogenic proteins including VEGF and SDF-1α [38]. Formation of the SDF-1α gradient leads to mobilization of EPCs. Further, chemotaxis of EPCs toward SDF-1α is increased by IL-3 and EPCs derived from the bone marrow respond better than those isolated from circulation [39]. The chemokine eotaxin or CCL11 mediates angiogenesis either directly via the CCR3 receptor of human microvascular endothelial cells or indirectly by recruitment and activation of eosinophils which release angiogenic factors like transforming growth factor α and β (TGF-α, TGF-β) [22]. Chemokine CXCL1 and its receptor CXCR2 are involved in endothelial repair after injury. Recently, activated platelets have been implicated in EPC recruitment and migration via release of β-thromboglobulin, a precursor CXCL12 and CXCL7 [15].

Recent studies involving endothelial nitric oxide synthase have showed that nitric oxide (NO) plays an important role in angiogenesis involving mature endothelial cells and neovasculogenesis involving EPCs [40]. In models of mice deficient in endothelial nitric oxide synthase (NOS3^-/-^), VEGF stimulation of EPC mobilization was reduced and only intravenous infusion of wild type progenitor cells, not bone marrow transplantation, resulted in restoration of neovascularization, demonstrating the role of nitric oxide in mobilization of progenitor cells into circulation [41]. In rat bone marrow ex vivo models, administration of angiotensin II lead to eNOS dependent NO production in EPCs and modulated EPC adhesion and apoptosis [42].

Exogenous factors, including drugs like Statins and Thiazolidinediones are also involved in EPC migration and proliferation. Statins are drugs which inhibit the enzyme 3-hydroxy-3-methylglutryl coenzyme A (HMG-CoA) involved in cholesterol biosynthesis. They also activate both endothelial progenitor cells and mature endothelial cells by stimulation of the Akt signalling pathway [43]. Endogenous factors used therapeutically like G-CSF and GM-CSF, used to treat haematological diseases, are known to induce BM-EPCs mobilization and migration and may present further complications. The factors listed above utilize prosurvival chemokine/cytokine mediators for cellular modulation. We and others discovered the presence of estrogen receptor in EPCs suggesting a novel role of the E2-ER pathway in the survival and biological activities of EPCs.

## Role of ER on EPC neovascularization

Epidemiological observations have indicated a role of hormones, specifically estrogen, in vascular repair and maintenance. Such observations include a comparative decrease of heart disease and increase in vascular repair in women compared to men. Initial work focused on the role of estrogen in ischemic tissue and heart models found that estrogen is indeed cardio protective and aids in vascular repair. One such mechanism is via upregulation of prostacyclin in endothelial cells leading to vasodilation and inhibiting platelet aggregation [44,45]. *In vivo *studies using estrogen receptor α and β knockout mice have verified that estrogen and its receptors are important specifically in EPC dependent neovascularization of ischemic tissue. The activation of EPCs by estradiol is predominantly mediated via ERα, and EPCs treated with estradiol showed an increased expression of ERα mRNA transcripts. Further, VEGF expression was increased in treated WT EPCs whereas VEGF expression was minimal in ERα knock out EPCs [46]. Estrogen activates EPCs via the PI3K/Akt pathway, phosphotidlyinositol-3 kinase (PI3K) converts PIP2 to PIP3, PIP3 in turn phosphorylates Akt which is responsible for EPC migration and proliferation [47]. Estrogen also increases the telomerase activity in EPCs and prolongs their survival [48]. Interestingly, in mice deficient for eNOS expression, estradiol has no effect of EPC mobilization, indicating a major role of nitric oxide in EPC function [49]. Estrogen also exerts effects on non-ischemic EPC aided vascularization, for example previous work observed a cyclical increase in EPC mobilization following a rise in estrogen and VEGF levels during menstrual cycling in uterine tissue [50,51].

Recently, the role of estrogen in tumor induced neovascularization has emerged lending focus to its ability to significantly impact not only tumor growth and development but also metastasis. Previously, our lab observed an increase in BM-EPC mobilization and homing to tumor tissue in an *in vivo *transgenic mouse breast tumor model when mice were supplemented with a slow release estradiol pellet. This supplementation lead not only to an increase in tumor vessel formation but also an increase in mRNA transcripts of proangiogenic genes including angiopoietins 1 and 2, MMPs 2 and 9, and VEGF [5]. Using transgenic animals in which GFP was under control of the Tie2 (TEK) promoter, we were able to visualize BM-EPC association with tumor blood vessels. Further, in an *in vitro *model tumor cell conditioned media from estradiol supplemented cells also lead to BM-EPC tubulogenesis when compared to control conditioned media [5]. Thus, hormones, in particular estrogen, play a large role in EPC function and are pivotal in tumor development in hormone responsive tissues. It is this novel mechanism of estrogen mediated tumor progression that will be the aim of future therapeutic strategies.

## Potential for future work

While the major physiological role of circulating EPCs in adults is to maintain vascular integrity, they can also home to and aid in revascularization of ischemic and tumor tissues [7]. Indeed previous clinical correlations have reported an increase in EPC circulation in breast, ovarian and pancreatic cancer patients with a positive correlation to tumor stage and size [6,52,53]. It is this observation that may prove EPC's usefulness as a biomarker for early tumor detection where EPCs serve as a sensor of tumor initiation. Further, tagging of EPCs may allow tracing of their mobilization and homing to tumor tissues aiding in specific, targeted early detection of tumor growth, a critical determinant of aggressive tumor growth outcome.

This targeted homing can be manipulated for future therapeutic research. One such method may utilize EPCs as gene delivery vehicles in the treatment of tumors. Such a method would involve *ex vivo *expanded EPCs that can be transduced with a transgene expressing anti-angiogenic factors and administered to patients directed at blocking tumor growth [54]. Drug delivery vehicles currently used to deliver chemotherapeutic drugs to the tumors are liposomes and exosomes, analogous to these, EPCs can be used as a 'Trojan horse' for targeted delivery of drugs to tumor tissues. Another potential therapeutic strategy aimed at blocking EPC mobilization and migration from the bone marrow itself would also impact tumor growth and metastasis and may increase efficacy of early detection and surgical intervention [20].

While the methods described may prove EPCs as powerful weapons against cancer development, their role in other physiological functions also needs consideration. EPCs have a possible therapeutic benefit in ischemic diseases as injection of *ex vivo *expanded EPCs into patients may have potential regenerative effects in ischemic tissues opening the door to novel treatment strategies for diabetes. EPCs may also be used to construct endothelial coated vascular grafts which may have a better patency rate [55]. On the negative side increasing the number of circulating EPCs to promote neovasculogenesis in ischemia should be investigated for plaque destabilization and differentiation into atherogenic cells which can cause embolism [56].

## Conclusions

Endothelial Progenitor cells originate from the bone marrow and have the ability to differentiate into multiple cell lines. Endogenous factors like VEGF, cytokines, estradiol, and eNOS with exogenous factors like statins and thiazolidinediones mediate recruitment of EPCs into the circulation. Circulating EPCs have a wide array of functions in tissue regeneration, tissue remodelling and cancer progression. In tumors and ischemic tissues EPCs have a direct structural role of differentiating into mature endothelial cells and an indirect paracrine effect by secreting angiogenic factors. Hypoxia in ischemic tissues and during the early phase of tumor growth is crucial for EPC recruitment and is mediated via upregulation of HIF-1 leading to an increase in the transcription of proangiogenic proteins including VEGF. EPCs also play a major role in the pathogenesis of heart failure, diabetes and vascular diseases with studies showing that high circulating EPCs have a direct correlation with decreased vascular complications. Further research to study the biology of EPCs is essential and ultimately will lead to the development and utilization of EPCs as a powerful diagnostic, therapeutic and prognostic tool in a wide variety of diseases.

## List of abbreviations used

BM-EPCs: bone marrow-derived endothelial progenitor cells; IL-1: interleukin 1; TGF-α/β: transforming growth factor alpha/beta; EMT: epithelial to mesenchymal transition; SA: sprouting angiogenesis; IA: intussesceptive angiogenesis; VEGF: vascular endothelial growth factor; VEGFR: vascular endothelial growth factor receptor; MAPCs: multipotent adult progenitor cells; vWF: vonWillebrand factor; LDL: low-density lipoprotein; UEA1: *Ulex europaeus *agglutinin 1; KDR: kinase insert domain protein receptor; MNCs: mononuclear cells; EOCs: endothelial outgrowth cells; PSGL-1: P-selectin glycoprotein ligand-1; LFA-1: lymphocyte function-associated antigen 1; FGF: fibroblast growth factor; SDF-1: stromal derived factor 1; mKitL: membrane-crossing Kit ligand; sKitL: solubility Kit ligand; MCP-1: monocyte chemoattractant protein-1; MRI: magnetic resonance imaging; HIF-1: hypoxia inducible factor 1; MMP: matrix metalloproteinase; G-CSF: granulocyte-colony stimulating factor; GM-CSF: granulocyte monocyte-colony stimulating factor; HUVECs: human umbilical vein endothelial cells; VSMCs: vascular smooth muscle cells; RANTES: regulated upon activation, normal T-cell expressed and secreted; NO: nitric oxide; NOS3 (eNOS): nitric oxide synthase 3 (endothelial nitric oxide synthase); HMG-CoA: 3-hydroxy-3-methylglutryl coenzyme A; ERα: estrogen receptor alpha; PI3K: phosphatidylinositol 3-kinases; PIP2: phophatidylinositol bisphosphate; PIP3: phophatidylinositol (3,4,5)-triphosphate; WT: wild type

## Competing interests

The authors declare that they have no competing interests.

## Authors' contributions

ALG, PBP, SR, RS, AS, AM, RKT involved in concept design, coordination, drafting and critically revising the manuscript and have given final approval.
